# Oral Administration of *Lactococcus lactis* Subsp. *lactis* JCM5805 Enhances Lung Immune Response Resulting in Protection from Murine Parainfluenza Virus Infection

**DOI:** 10.1371/journal.pone.0119055

**Published:** 2015-03-06

**Authors:** Kenta Jounai, Tetsu Sugimura, Konomi Ohshio, Daisuke Fujiwara

**Affiliations:** 1 Technical Deveropment Center, Koiwai Dairy Products Co Ltd. Sayama, Japan; 2 Central Laboratories for Key Technologies, Kirin Co. Ltd., Yokohama, Japan; Imperial College London, UNITED KINGDOM

## Abstract

When activated by viral infection, plasmacytoid dendritic cells (pDCs) play a primary role in the immune response through secretion of IFN-α. *Lactococcus lactis* subsp. *lactis* JCM5805 (JCM5805) is a strain of lactic acid bacteria (LAB) that activates murine and human pDCs to express type I and type III interferons (IFNs). JCM5805 has also been shown to activate pDCs via a Toll-like receptor 9 (TLR9) dependent pathway. In this study, we investigated the anti-viral effects of oral administration of JCM5805 using a mouse model of murine parainfluenza virus (mPIV1) infection. JCM5805-fed mice showed a drastic improvement in survival rate, prevention of weight loss, and reduction in lung histopathology scores compared to control mice. We further examined the mechanism of anti-viral effects elicited by JCM5805 administration using naive mice. Microscopic observations showed that JCM5805 was incorporated into CD11c^+^ immune cells in Peyer’s patches (PP) and PP pDCs were significantly activated and the expression levels of IFNs were significantly increased. Interestingly, nevertheless resident pDCs at lung were not activated and expressions levels of IFNs at whole lung tissue were not influenced, the expressions of anti-viral factors induced by IFNs, such as *Isg15*, *Oasl2*, and *Viperin*, at lung were up-regulated in JCM5805-fed mice compared to control mice. Therefore expressed IFNs from intestine might be delivered to lung and IFN stimulated genes might be induced. Furthermore, elevated expressions of type I IFNs from lung lymphocytes were observed in response to mPIV1 *ex vivo* stimulation in JCM5805-fed mice compared to control. This might be due to increased ratio of pDCs located in lung were significantly increased in JCM5805 group. Taken together, a specific LAB strain might be able to affect anti-viral immunological profile in lung via activation of intestinal pDC leading to enhanced anti-viral phenotype *in vivo*.

## Introduction

Probiotics are live microorganisms in intestinal flora or starter cultures for dairy products that have been reported to have beneficial effects on human health. Lactic acid bacteria (LAB) had been reported to produce potent and diverse immunomodulatory effects. Among these, the protective effect of LAB against viral infection is of particular interest. For example, oral administration of *Bifidobacterium breve* YIT4064 has been shown to be effective against rotavirus-induced diarrhea and influenza virus infection [1,2]. *Lactobacillus pentosus* S-PT84, *L*. *plantarum* 06CC2, *L*. *acidophilus* L-92, *Enterococcus faecalis* FK-23 and *L*. *casei* shirota have also been reported to protect mice from influenza virus infection [3–8]. However, little is known about the mechanism of the effect of LAB on viral infection.

Plasmacytoid dendritic cells (pDCs) act in the innate immune system as the first line of defense against viral infection and, triggered by viral nucleic acids, secrete a large amount of interferon-α (IFN-α) [9,10]. Recently, pDCs have been shown to be important not only as a producer of IFNs but also as a regulatory cell that control various immune subsets, such as CD4^+^ / CD8^+^ T cells and B cells [11,12]. Takagi et al. have shown that pDCs suppress induction of the CD4^+^ T cell response and participate in initiation of CD8^+^ T cells against virus, using Siglec-H-deficient mice and pDCs-ablated mice [13]. pDCs also play a prominent role in mucosal immunoglobulin A (IgA) productions by expression of APRIL and BAFF [14]. Therefore, stimulation of host pDCs is considered to have a protective effect against viral infection.

Although some pathogenic bacteria (e.g., *Staphylococcus aureus*) have been shown to stimulate pDCs [15], beneficial bacteria (e.g., LAB) have been shown to be ineffective in stimulating pDCs [16]. However, we screened non-pathogenic LAB strains and found that LAB strain JCM5805 stimulated murine pDCs to produce Type I and III IFNs in association with myeloid dendritic cells [17]. JCM5805 was also shown to activate human pDCs isolated from peripheral blood mononuclear cells (PBMCs) *in vitro* and administration of JCM5805 significantly affected pDC activity in humans [18].

Murine parainfluenza virus type 1 (mPIV1) is a member of the family *Paramyxovirida*: enveloped, negative-strand, single-stranded RNA (ssRNA) viruses. mPIV1 ssRNA is recognized by Toll-like receptor (TLR) 7 and TLR8 [19], which are highly expressed in pDCs, and the RIG-I helicase in the cytosol recognizes viral nucleic acids in conventional dendritic cells (cDCs) and fibroblasts [20–22]. mPIV1 has been reported to induce acute lung inflammation in mice, that are widely used as a respiratory viral infection model [23]. Because of its robust proliferation in embryonated eggs and cell cultures, mPIV1 has been extensively studied [24]. mPIV1 is highly infectious and induces pathological lesions in the lung, leading to lethality in mice [25].

Using the mPIV1-infected mouse model, we studied the preventive effects of oral administration of JCM5805 against viral infection. We also investigated the mechanism of activation of anti-viral immunity induced by oral administration of JCM5805 by examining immunity in intestine and lung tissues using naive mice. As a result, we found that oral administration of JCM5805 protected mice against mPIV1 infection by activating an immune response in lung tissue by stimulating pDCs localized in the intestine.

## Materials and Methods

### Mice

For studies of the anti-viral effects of JCM5805 administration in mPIV1-infected mice, 6- to 10-weeks-old female wild-type DBA/2JJcl mice were purchased from CLEA Japan. DBA/2JJ mice are used for experiment of mPIV1 infection, because its susceptibility against mPIV1. Mice were divided equal average weight into two groups. Control group mice (n = 18) were fed AIN93G (Oriental Yeast, Tokyo, Japan) and JCM5805 group mice (n = 19) were fed AIN93G containing of 1 mg heat-killed JCM5805 / day / mouse and water ad libitum. The mice were housed three per cage in specific pathogen-free conditions under a 12hr light / dark cycle. The temperature in the room was kept at 22 ± 2°C and 60 ± 15% humidity. The treatment was started 14 days before mPIV1 infection and continued for 15 days after infection. Animal procedures and experiments were approved by the Laboratory Animal Care Committee of the Central Institute for Experimental Animals. The approval ID of these experiments was CA1101.

For studies of the effect of JCM5805 administration on immunity in naive mice, 8- to 10-weeks old female C57BL/6J wild-type mice were purchased from Charles River Japan. Mice were divided equal average weight into two groups. Control group mice (n = 8) were fed AIN93G and JCM5805 group mice (n = 8) were fed AIN93G containing of 1 mg heat-killed JCM5805 / day / mouse and water ad libitum. The mice were housed one per cage in specific pathogen-free conditions under a 12hr light / dark cycle. The temperature in the room was kept at 25 ± 1°C and 60 ± 15% humidity. Animal procedures and experiments were approved by the Laboratory Animal Care Committee of Central Laboratories for Key Technologies, Kirin Co., Ltd. The approval ID of these experiments was YO11-00147. Adequate measures were taken to minimize pain and discomfort taking into account human endpoints for animal suffering and distress. Animals were monitored for their conditions and the clinical scores were recorded every day after the mPIV1 infection. Animals surviving the infection were sacrificed anaesthetically at day 15 using diethyl ether.

### mPIV1 infection

mPIV1 was prepared by the Central Institute for Experimental Animals. Mice were inoculated twice using a micropipette, with 4 hr between inoculations, by intranasal administration of a 25 μl drop containing 64 hemagglutination units (HAU) of mPIV1.

### Body weight measurements

Net body weights were measured daily during the course of experiments of mPIV1 infection. Animals surviving the infection were weighed daily and those with severe weight loss of 25% or more were sacrificed anesthetically.

### General conditions and number of mice with emaciation after infection with mPIV1

General conditions and unusual condition of the animals were recorded daily by clinical observation. Number of mice with emaciation was evaluated every day after the mPIV1 infection for 15 days. The examination was performed by two independent observers with the same observation criteria to eliminate possible bias associated with individuals. The emaciation state was evaluated as following features: −: no emaciation; +: emaciation. Once the animals display humane endpoints, they are humanely killed.

### Lung histopathology

At 3 days post-infection, six mice each from the control and JCM5805 groups were sacrificed anesthetically and their lungs were taken from the mice the right lung was taken from six mice and fixed with 10% PFA. Fixed sections of paraffin-embedded lungs were stained with hematoxylin and eosin (H&E) (Sakura Finetek). Slides were randomized, read blindly, and examined for tissue damage and inflammatory cellular infiltration. The observations were scored in four levels as follows: 0, no pathogenicity; 1, low pathogenicity; 2, medium pathogenicity; 3, high pathogenicity.

### Microscopic observation of phagocytosis in Peyer’s patches

Fluorescent-labeled JCM5805 were prepared as follows: 30 mg JCM5805 / 1 ml FITC solution [0.1 mg FITC isomer 1 (Sigma) / ml 0.1 M NaHCO_3_ buffer, pH 9.0] was incubated for 60 min at 25°C and then washed three times with PBS. To investigate phagocytosis by CD11c^+^ cells, C57BL/6J mice were orally administered FITC-labeled JCM5805 (30 mg/mouse), sacrificed anaesthetically 8 hr later, and their Peyer’s patches (PP) were excised. PP cells were frozen in Tissue-Tek O.C.T. compound (Sakura Finetek, Torrance, CA) and sliced into 6 μm sections. The sections were fixed in cold acetone (Wako) for 10 min at −20°C. Then the slides were washed with PBS containing 1% BSA and stained with affinity purified anti-CD11c (Thermo) for 2 hr at room temperature. The slides were washed again with PBS and stained with anti-rat IgG labeled with Alexa Fluor 546 (Invitrogen) for 30 min at room temperature. The slides were washed again with PBS and mounted with Fluoromount (Diagnostic Biosystems). Fluorescence microscopy was performed using an Olympus BX60.

### Intestinal cell preparation and FACS analysis

PP were minced in Mg^2+^- and Ca^2+^-free Hank’s Balanced Salt Solution and digested with 1 mg collagenase (Sigma) / ml and 0.2 mg DNase I / ml for 20 min at 37°C. EDTA was added to 30 mM and the mixture was incubated for 10 min at room temperature. Tissue lysates were filtered through a 70 μm nylon cell strainer and layered onto 15% Histodenz (Sigma) in RPMI 1640 containing 10% FCS, and centrifuged at 450 x g for 20 min without braking. The low density fractions at interfaces were collected and washed. The cells were stained with a fluorescent dye conjugated to an antibody as follows: I-A / I-E-FITC (M5 / 114.15.2) (eBiosciencs), CD3ε-PE (145-2C11) (eBiosciencs), 7-AAD (BD Pharmingen), Siglec-H-APC (551.3D3) (Milteny Biotec), and CD11c-PE-Cy7 (N418) (eBiosciencs). After staining, the cells were washed twice with FACS buffer (0.5% BSA in PBS buffer) and suspended in 4% paraformaldehyde (Wako) for FACS analysis. Data were collected by a FACS Canto II (BD Biosciences) and analyzed by FCS Express software (De Novo Software). CD3^−^ CD11c^+^ Siglec-H^+^ cells were defined as pDCs, and the expression levels of cell surface markers on these pDCs were measured.

### Gene expression analysis

Total RNA was extracted using an RNeasy Kit (Qiagen), and cDNA was prepared using an iScript cDNA synthesis kit (BioRad), according to the manufacturer’s protocol. Quantitative RT-PCR (qRT-PCR) was performed using SYBR Premix Ex Taq (TaKaRa) and a LightCycler 480 (Roche). The methods and primers for qRT-PCR of *Gapdh*, *Ifnα*, *Ifnβ*, *Isg15*, *Oasl2*, *Viperin* and *Cxcl12* have been previously described [26,27]. mRNA expression of GAPDH was used as internal control for normalization of gene expression analysis. The nucleotide sequences of primers were follows: *Gapdh* forward (F) (AACGACCCCTTCATTGAC) and *Gapdh* reverse (R) (TCCACGACATACTCAGCAC), *Ifnα* F (AGCAGGTGGGGGTGCAGGAA) and *Ifnα* R (ACCACCTCCCAGGCACAGGG), *Ifnβ*F (TCAGAATGAGTGGTGGTTGC) and *Ifnβ* R (GACCTTTCAAATGCAGTAGATTCA), *Isg15* F (GAGCTAGAGCCTGCAGCAAT) and *Isg15* R (TTCTGGGCAATCTGCTTCTT), *Oasl2* F (CGATGCCTGGGAGAGAATCG) and *Oasl2* R (TCGCCTGCTCTTCGAAACTG), and *Viperin* F (CTTCAACGTGGACGAAGACA) and *Viperin* R (GACGCTCCAAGAATGTTTCA), and *Cxcl12* F (GAGCCAACGTCAAGCATCTG) and *Cxcl12* R (CGGGTCAATGCACACTTGTC).

### Expression of IFNs against inactivated mPIV1 *ex vivo*

Single-cell suspensions of lung samples were prepared as previously described [28]. Lung lymphocytes were cultured at a density of 1 × 10^6^ cells / ml RPMI medium in 48 well plates for 24 hr at 37°C, with or without 0.5 AU inactivated mPIV1 / ml. The concentrations of IFN-α and IFN-β in culture supernatants were analyzed by ELISA (PBL Biomedical Laboratories).

### Statistics

Statistical differences between two groups were determined using an unpaired, two-tailed Student’s t test with significance set at P<0.05. For survival studies, a Log-Rank (Mantel-Cox) test was used. Clinical scores were evaluated using a chi-square test. For lung histopathology, the Mann-Whitney U test was used.

## Results

### Effect of JCM5805 on mPIV1 infection

**Fig 1:**
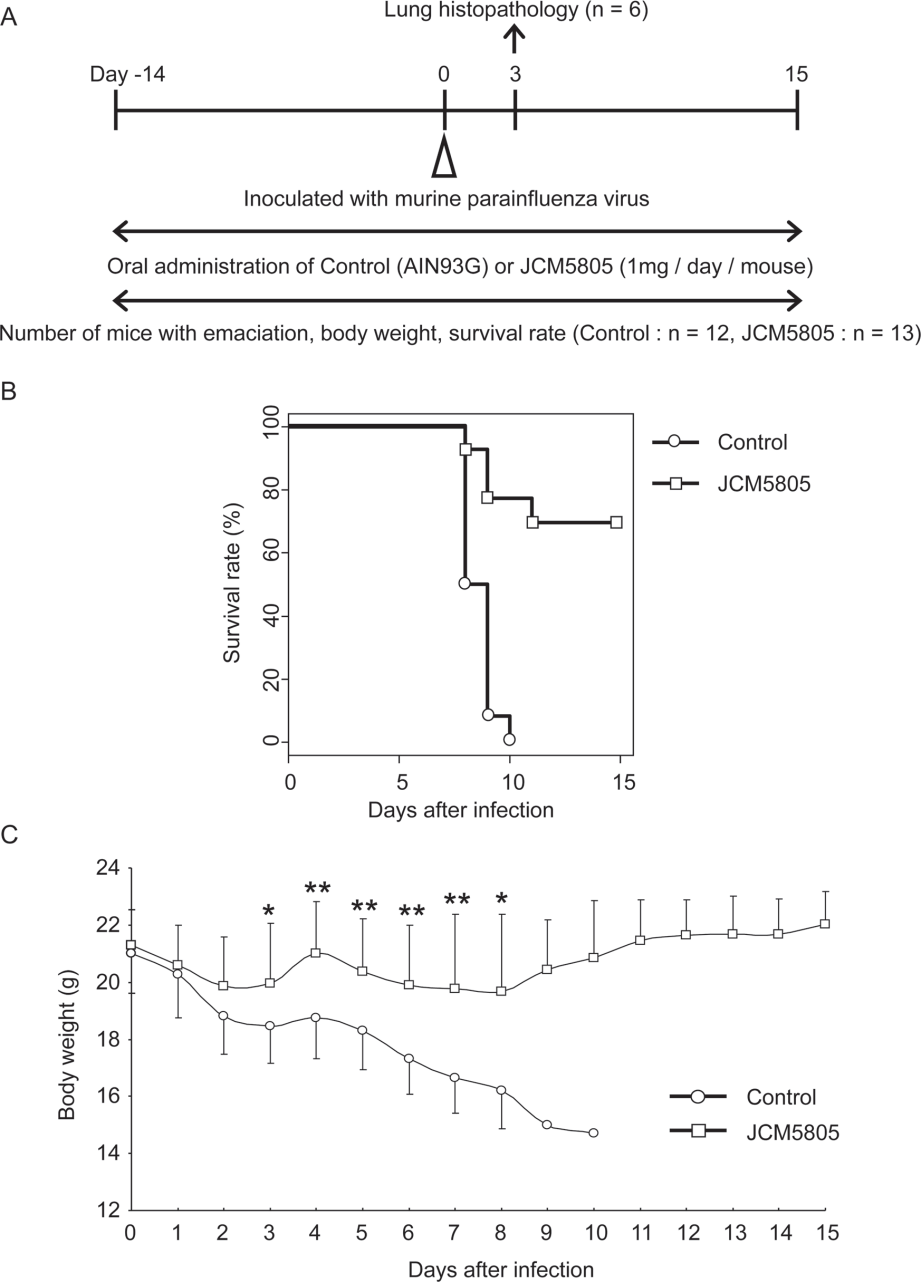
Effects of JCM5805 on mPIV1 infection. A. Experimental procedure of mPIV1 infection. Mice in the control and JCM5805 groups were fed diet with or without 1 mg / mouse / day of JCM5808 during the study period (day -14 to 15). Mice were intranasally infected with mPIV1 on day 0. On 3 days post-mPIV1 infection, six mice were sacrificed from each group for lung histopathology. Thereafter survival rate, body weight and clinical scores were investigated with remained control mice n = 12, and JCM5805 mice n = 13. B. Survival rate of mice infected with mPIV1. The control (circle) and JCM5805 (square) groups consisted of 12 and 13 mice, respectively. The survival of each animal was monitored daily. *P*<0.001 (Log-Rank test). C. Body weight of mice infected with mPIV1. The control (circle) and JCM5805 (square) groups consisted of 12 and 13 mice, respectively. The body weight of each surviving animal was measured daily. The body weight values are shown as mean ± SD. *P<0.05, **P<0.01 (Student’s t test). The data shown is representative of two independent experiments.

mPIV1 infection of the mouse lung causes pathological lesions leading to lethality [25]. The mouse mPIV1 infection model was used to study the effect of JCM5805 on viral infection by feeding a diet containing JCM5805 starting 14 days before mPIV1 infection (Fig. 1A). Six mice from the control and JCM5805 groups were sacrificed anesthetically 3 days after infection and the lungs were isolated to examine histopathology.

Fig. 1B shows the survival rates of mice in the control and JCM5805 groups up to 15 days after mPIV1 infection. Animals surviving the infection were weighed daily and those animals with severe weight loss of 25% or more were sacrificed anesthetically and regarded as dead animals. The survival rate of control group mice was 50.0% at day 8, 8.3% at day 9, and 0% at day 10, whereas for JCM5805 group mice it was 92.3% at day 8, 76.9% at day 9, and 69.2% at day 11. Therefore, there was a drastic increase in the survival rate (P<0.001) of JCM5805 group mice compared to control group mice.

As shown in Fig. 1C, the body weight of mPIV1-infected control group mice showed a continuous decrease throughout the experiment. In contrast, the body weight of mPIV1-infected JCM5805 group mice decreased slightly for a few days after infection, then leveled off for a few days and finally increased slightly through the end of the experiment.

Table 1 shows the number of mice with emaciation after infection. JCM5805 group mice showed significantly lower number of mice emaciation after day 6 compared to control group.

**Table 1:** Numbers of mice with emaciation after infection with mPIV1.

Days after infection	Group	Number of mice with emaciation		Chi-square test
		−	+	
3	Control	11	1	P = 0.288
	JCM5808	13	0	
4	Control	11	1	P = 0.288
	JCM5805	13	0	
5	Control	9	3	P = 0.548
	JCM5805	11	2	
6	Control	1	11	**P < 0.01
	JCM5805	11	2	
7	Control	0	12	**P < 0.01
	JCM5805	10	3	
8	Control	0	6	**P < 0.01
	JCM5805	9	3	
9	Control	0	1	*P < 0.05
	JCM5805	9	1	
10	Control	0	1	*P < 0.05
	JCM5805	9	1	

The emaciation state of mice in the control and JCM5805 groups were evaluated after mPIV1 infection. The emaciation state was evaluated as follows: −, no emaciation; +, emaciation.

*P<0.05

**P<0.01 (Chi-square test).

The data shown is representative of two independent experiments.

**Fig 2:**
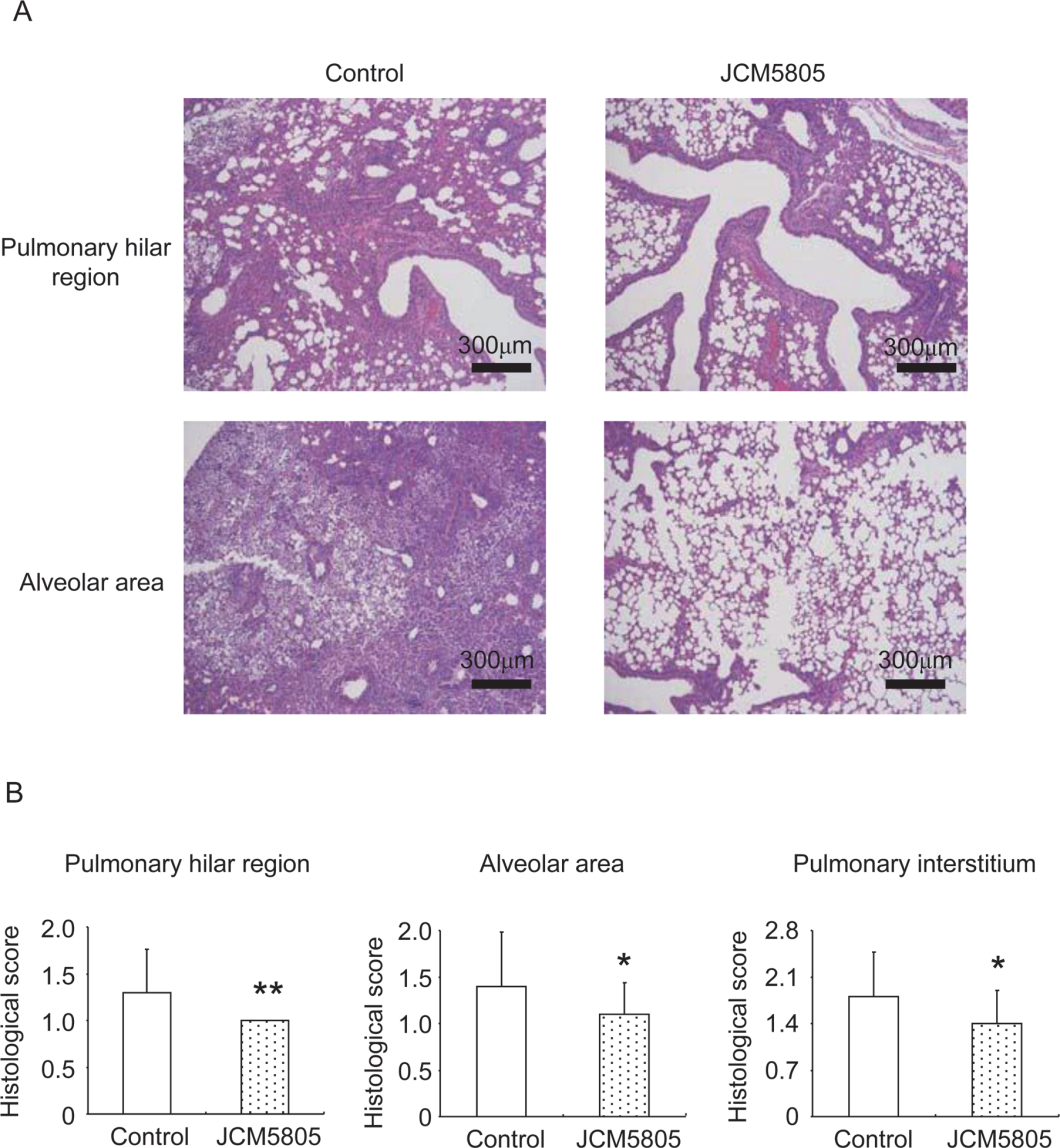
Lung histopathology of mPIV1-infected mice. A. Representative hematoxylin and eosin (H & E)-stained sections of lung tissues from control and JCM5805 group mice (6 mice per group). Lung tissues were prepared from mice 3 days after infection. Scale bars, 300 μm. B. Histological scoring of lung tissues from mPIV1-infected mice belong to control (open columns) and JCM5805 (dot columns) group. Sections were scored at four levels as follows: 0, no symptoms; 1, low pathogenicity; 2, medium pathogenicity; 3, high pathogenicity. The mean ± SD of the tissues in each group is shown. *P<0.05, **P<0.01 (Mann-Whitney U test). The data shown is representative of two independent experiments.

Fig. 2A shows microscopic fields of lung tissue from mPIV1-infected mice in each group. Control group mice had extensive lung damage; i.e., highly basophilic epithelium lining the bronchioles, focal degenerating cells undergoing apoptosis or necrosis, and extensive cellular infiltrates of neutrophils, monocytes and lymphocytes. However, JCM5805 group mice showed decreased prevalence of epithelial cells with morphologic features of degeneration and necrosis (Fig. 2B). These data strongly indicated that oral administration of JCM5805 before mPIV1 infection was highly effective in preventing and / or reducing viral pathogenicity.

### Fluorescence microscopy of JCM5805 incorporation into PP

**Fig 3:**
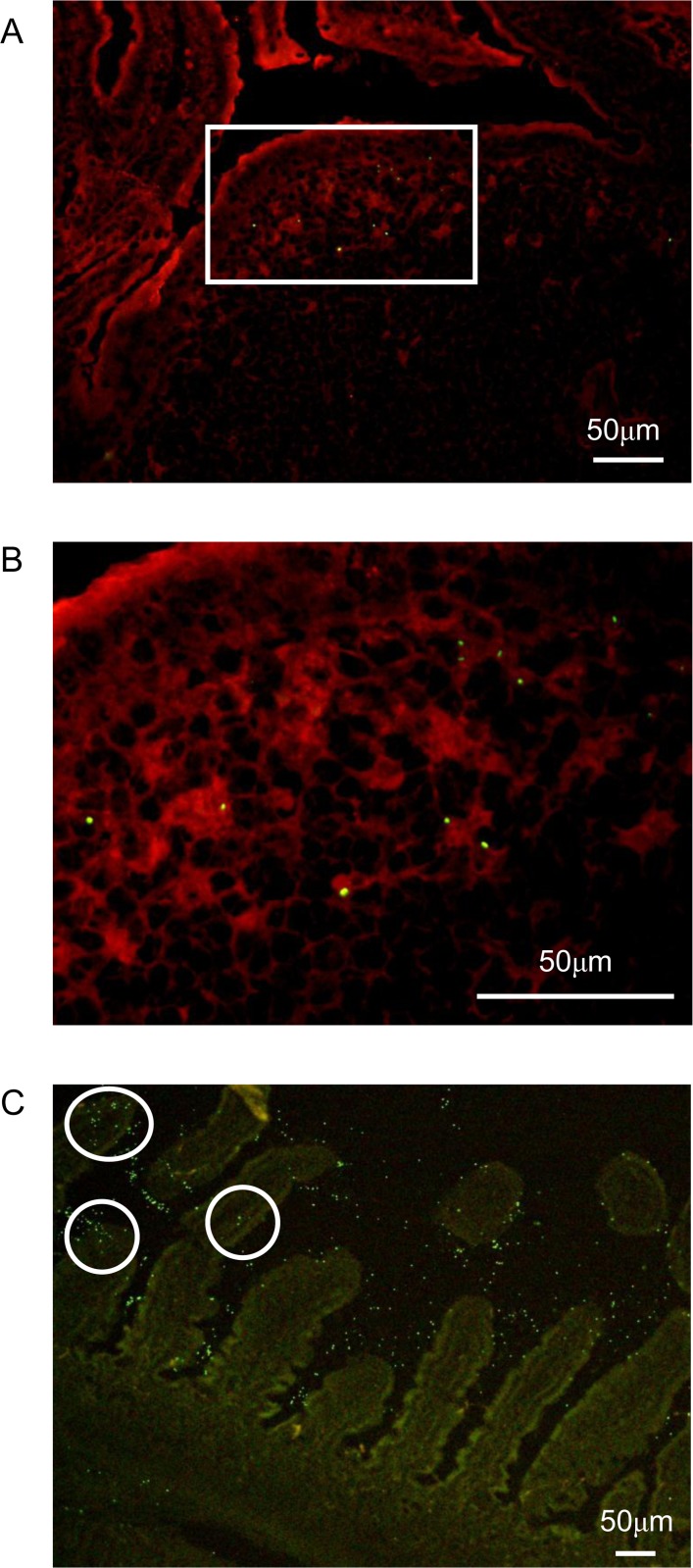
Fluorescence microscopy observation of intestine derived from JCM5805-administered mice. PPs were removed 8 hr after oral administration of FITC-labeled JCM5805 (green). (A, B) Observations at PP sections where CD11c was labeled with anti-CD11c (red). Magnifications of microscopic images are (A) × 200 and (B) × 600. (C) Observation at intestinal villi. The original magnification is × 100. These data are representative from two experiments that yielded similar results.

We then used naive mice to investigate the mechanism by which JCM5805 may affect viral pathogenicity. FITC-labeled JCM5805 was orally administrated to mice and frozen sections of PP were stained by CD11c antibody and visualized by fluorescence microscopy. As shown in Fig. 3A and B (magnified image of white boxed area of Fig. 3A), FITC-labeled JCM5805 was incorporated into CD11c^+^ immune cells in the subepithelial dome (SED) area and was also found inside the small intestinal lamina propria (Fig. 3C).

### Activation of pDCs in intestine by JCM5805 administration

**Fig 4:**
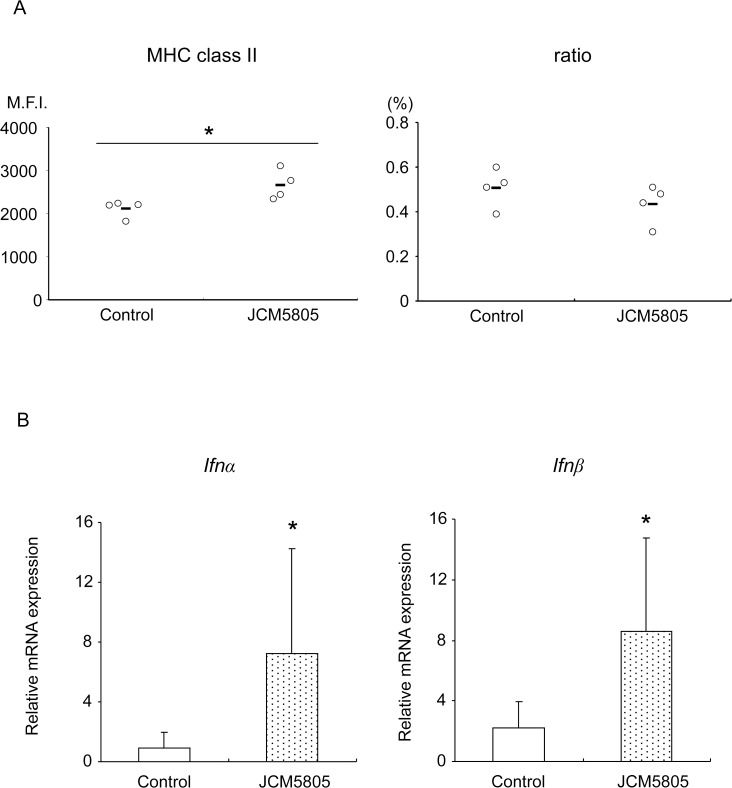
Activation of pDCs in intestine by JCM5805 administration. Healthy C57BL / 6J mice were divided into control and JCM5805 groups (n = 4 in each group), and mice in the JCM5805 group were orally administered JCM5808 daily for 2 weeks. A. Low density cells prepared from PP of each group were analyzed by FACS. Expression level of cell surface activation marker was evaluated for MHC class II as median fluorescence intensities (M.F.I.) in left panel. Ratio of pDCs to total population was shown in right panel. pDCs was defined as “CD3^−^ Siglec-H^+^ CD11c^+^ in total population”. Short line represents the mean values. *P<0.05 (Student’s t test). B, Total mRNA was extracted from PP pDCs from mice in the control (open columns) and JCM5805 groups (dot columns) (n = 8 in each group). *Ifnα* and *Ifnβ* gene expressions were measured by qRT-PCR and normalized to *Gapdh* gene expression. Data are shown as mean ± SD. *P<0.05 (Student’s t test). These data are representative of three independent experiments. Each data are mean ± SD.

To examine the activation of pDCs in PP after JCM5805 incorporation, status of pDCs were analyzed in PP by FACS two weeks after JCM5808 administration. Expression of MHC class II on pDCs in JCM5808 group mice was significantly greater than in control group mice (Fig. 4A). However, ratio of pDCs in JCM5805 group mice was not significantly affected compared to control group. We next examined expression level of type I IFNs from pDC located in PP. Both *Ifnα* and *Ifnβ* mRNA levels in PP pDCs prepared from JCM5805 group were significantly greater than control group (Fig. 4B). These data suggested that JCM5805 was taken up from the intestinal tract through PP and activated resident pDCs to increase type I IFNs at draining mucosal sites.

### Effects of JCM5805 on anti-viral immunity at lung

**Fig 5:**
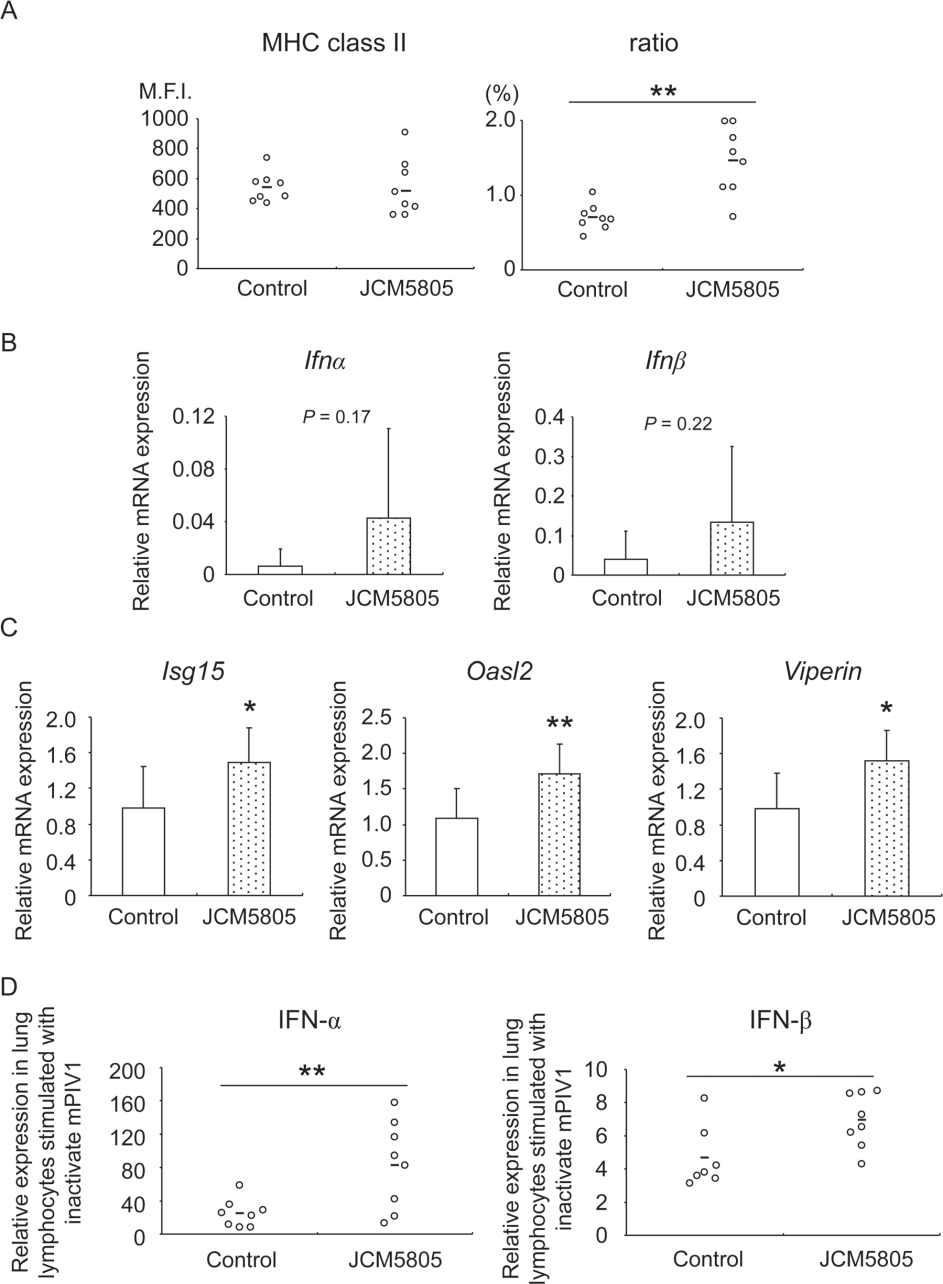
Effects of JCM5805 administration on lung immunity. Healthy C57BL / 6J mice were divided into control and JCM5805 groups (n = 8 in each group), and then mice in the JCM5805 group were orally administered JCM5808 daily for 2 weeks. A. Low density cells prepared from lung of each group were analyzed by FACS. Expression level of cell surface activation marker was evaluated for MHC class II as M.F.I. in left panel. Ratio of pDCs to total population was shown in right panel. pDCs was defined as “CD3^−^ Siglec-H^+^ CD11c^+^ in total population”. Short line represents the mean values. **P<0.01 (Student’s t test). B. Total mRNA was extracted from lung tissues from control (open columns) and JCM5805 (dot columns) group. *Ifnα* and *Ifnβ* gene expressions were evaluated by qRT-PCR normalized by *Gapdh*. C. Total mRNA was extracted from lung tissues from mice in the control (open columns) and JCM5805 (dot columns) groups. *Isg15*, *Oasl2*, and *Viperin* gene expressions were estimated by qRT-PCR and normalized to *Gapdh* gene expression. Data are shown as mean ± SD. *P<0.05, **P<0.01 (Student’s t test). D. Lung lymphocytes prepared from control and JCM5805 group mice were cultured with inactivated mPIV1 for 24 hr, and the concentrations of IFN-α and IFN-β were measured in the culture supernatants. Each dot is correspondent to individual mouse. Short line means the mean values. *P<0.05, **P<0.01 (Student’s t test). The data are representative of three independent experiments. Each data are shown as mean ± SD.

Little is known about the effect of orally administrated LAB on systemic and local immunity or about how host-microbe crosstalk affects the immune response in peripheral tissues, such as lung. To determine whether oral administration of JCM5805 affects resident pDCs at lung, pDCs status and IFNs-related gene expressions were evaluated using JCM5805-fed mice. FACS analysis revealed that the expression level of activation marker, MHC class II, was not changed. However, ratio of pDCs at lung tissue derived from JCM5805 group was increased compared to control (Fig. 5A). Next, expression level of IFNs were not changed between two groups when it was compared using RNA prepared from whole tissue due to limited number of pDCs at lung (Fig. 5B). However, interestingly, expressions level of IFN-related genes, *Isg15*, *Oasl2* and *Viperin* were greater in lung whole tissues of JCM5805 group compared to control group (Fig. 5C). These data may imply that expressed IFNs by PP pDCs after oral administration of JCM5808 might be dispersed throughout of the whole body including lung and as a consequence expression of IFN-related genes in lung might be induced. Furthermore we sought whether lung immune response against mPIV1 might be affected by JCM5805 administration, lung lymphocytes derived from control and JCM5805 group were cultured with inactivated mPIV1 *ex vivo* and the expressions of IFN-α and IFN-β were measured. As a result, both IFN-α and IFN-β expressed by lung lymphocytes from JCM5805 group were significantly elevated compared to control group (Fig. 5D). This might be reflected by increased pDCs ratio at lung in JCM5805 group. Taken together, it was strongly suggested that anti-viral immunity at lung was indeed affected by JCM5805 administration via pDCs activation at intestine.

## Discussion

Since direct activation of pDCs and induction of type I and III IFNs by LAB have been shown to be minimal [17], a mouse model of mPIV1 infection was used in this study to evaluate the antiviral effect of LAB JCM5805 administration. As a result, it was shown that a significant improvement, such as survival rate, body weight and histopathology scores of lung tissues, were observed in JCM5805 group compared to control.

A number of studies have reported that LAB have some beneficial anti-viral effects against influenza virus in mice [3–8], by oral administration of *L*. *plantarum* 06CC2 (10 mg/day/mouse) or *L*. *acidophilus* L-92 (10 mg/day/mouse), or intranasal administration of *L*. *pentosus* S-PT84 (20 or 200 μg/day/mouse) or *L*. *casei* shirota (20 or 200 μg/day/mouse). These anti-viral effects of LABs are consistent with activation of classical innate immunity, such as NK cells and macrophages.

pDCs have been shown to contribute to host anti-viral defense through multiple mechanisms. Not only pDCs secrete type I IFN, but also they secrete IL-12 to induce Th1 polarization of CD4^+^ T cells [29]. pDCs are also able to destroy virus-infected cells in a FasL- and TRAIL-dependent manner [30,31]. Recently, Takagi *et al*. have shown that pDCs are responsible for generation of virus-specific CD8^+^ T cells by using inducible ablation mice model of pDCs *in vivo* [13]. In addition, Swiecki *et al*. showed that pDCs play a crucial role in generating virus-specific CD8^+^ T cells and also in expansion of virus-specific NK cells in response to murine cytomegalovirus and vesicular stomatitis virus infection [32]. These reports strongly suggested that pDCs is important subset, not only in terms of early host response against virus infection as a member of innate immunity, which is a major source of type I IFN, but also in late host response by linking with virus-specific cytotoxic T cell expansion *in vivo*. Therefore, JCM5805 may be a unique and effective anti-viral therapy by stimulating both the adaptive and innate immune systems.

We also demonstrated that JCM5805 was taken up by CD11c^+^ cells in PP. The surface of PP is covered with specialized follicle-associated epithelia (FAE), where various antigens are captured by M cells [33]. Some *Lactobacillus* strains have been reported to be incorporated into CD11c^+^ and CD11b^+^ cells in PP [34,35], and JCM5805 was shown to be incorporated into CD11c^+^ cells in PP in this study. Recently, other routes, such as intestinal villous M cells [36] and CX3CR1^+^ DCs [37,38], have been reported to be novel antigen entry sites in the mucosal epithelium. In agreement with those reports, we also found that JCM5805 was taken up by the intestinal villus. Therefore, JCM5805 might stimulate intestinal pDCs using both PP and the intestinal villus as entry sites. DCs in PP can be divided into four populations: CD11c^high^ CD11b^+^ CD8α^−^ DCs (CD11b^+^ DCs), CD11c^high^ CD11b^−^ CD8α^+^ DCs (CD8α^+^ DCs), CD11c^high^ CD11b^−^ CD8α^−^ DCs (DNDCs) and pDCs [39]. In this study, oral administration of JCM5805 was shown to activate pDCs in PP and induce expression of type I IFNs. Although the role of PP pDCs in the immune response is not fully understood, it has been reported that pDCs in GALT enhance the suppressive efficacy of CD4^+^ CD25^+^ Treg generation and might contribute to immune tolerance and gut homeostasis [40]. Since we have shown that enhanced Treg generation is occurred by JCM5805-treated pDCs *in vitro* [17], oral administration of JCM5805 might also contribute to the maintenance of gut homeostasis *in vivo*.

mPIV1 has been reported to induce acute lung inflammation in mice, which has been used as a respiratory viral infection model [23]. One of the biological effects of IFNs is induction of expression of IFN-stimulated genes (ISGs) that are involved in inhibition of viral replication and release. Expressions of three important ISGs (*Isg15*, *Oasl2* and *Viperin*) were examined in this study. *Isg15* inhibits replication and release of influenza virus and type I herpes simplex virus from infected cells [41], *Oasl2* is a 2’-5’-oligoadenylate synthetase that activates RNase L and degrades viral RNAs [41–43] and *Viperin* is a multifunctional antiviral factor that inhibits the growth of DNA and RNA viruses; e.g., hepatitis C virus, cytomegalovirus, influenza virus and flaviviruses [44,45]. In this study, oral administration of JCM5805 increased expression of these three ISGs in lung tissue, which is distant from JCM5808 entry site. This local increase of expression of anti-viral factors at lung may have contributed to the drastic increase in survival rate of JCM5808 group in mPIV1 infection experiment. Recently, Ichinohe *et al*. showed that host commensal microbiota composition critically regulates the generation of virus-specific CD4^+^ and CD8^+^ T cells and the antibody response in lung tissue following respiratory influenza virus infection [46]. Abt *et al*. reported that intestinal microbiota induces IFN-β production from peritoneal and alveolar macrophages via phospholyration of STAT1 and, as a result, expression of ISGs in lung tissue was elevated in mice infected by influenza virus [47]. These reports and our observation might imply that there is a close association between bronchus-associated lymphoid tissue (BALT) and GALT. We observed statistically significant increased expression of *Ifnα* and *Ifnβ* in PP pDCs in JCM5805 group compared to control, however there were not statistically significant change in lung tissue (*Ifnα*, P = 0.17, *Ifnβ*, P = 0.22.) Therefore, IFNs produced in intestine might affect expression of ISGs in lung tissue via the blood circulatory system. Interestingly, the response of lung lymphocytes prepared from JCM5805 group against mPIV1 was up-regulated compared to control. We observed increased ratio in resident pDCs at lung by JCM5805 administration, therefore the increased response by lung lymphocytes derived JCM5805 group may due to increased number of pDCs located at lung. It is intriguing that chemoattraction in lung might be affected by JCM5805 administration, therefore expression of CXCL12, which is reported to involved in migration of pDCs [48], were examined at lung. However no change was observed in CXCL12 (data not shown), other molecules involved in pDC chemoattraction might be changed by JCM5805 administration.

In conclusion, oral administration of JCM5805 was shown to elicit a significant anti-viral response against respiratory viral infection via enhancing lung immune response through activation of pDCs in the intestine in this study. Since we are regularly exposed to potential infectious threats, safe and effective immunomodulatory agents are widely required. LABs are generally accepted as safe food agents, specific LABs that affect pDCs might be useful and novel immune adjuvant that is able to increase systemic immune response by activating intestinal pDCs.
